# Molecular Mechanisms of Endometriosis Revealed Using Omics Data

**DOI:** 10.3390/biomedicines11082210

**Published:** 2023-08-07

**Authors:** Seong Beom Cho

**Affiliations:** Department of Biomedical Informatics, College of Medicine, Gachon University, 38-13, Dokgeom-ro 3 Street Namdon-gu, Incheon 21565, Republic of Korea; sbcho1749@gachon.ac.kr

**Keywords:** endometriosis, high-throughput data, disease gene, gene ontology, biological pathway

## Abstract

Endometriosis is a gynecological disorder prevalent in women of reproductive age. The primary symptoms include dysmenorrhea, irregular menstruation, and infertility. However, the pathogenesis of endometriosis remains unclear. With the advent of high-throughput technologies, various omics experiments have been conducted to identify genes related to the pathophysiology of endometriosis. This review highlights the molecular mechanisms underlying endometriosis using omics. When genes identified in omics experiments were compared with endometriosis disease genes identified in independent studies, the number of overlapping genes was moderate. However, the characteristics of these genes were found to be equivalent when functional gene set enrichment analysis was performed using gene ontology and biological pathway information. These findings indicate that omics technology provides invaluable information regarding the pathophysiology of endometriosis. Moreover, the functional characteristics revealed using enrichment analysis provide important clues for discovering endometriosis disease genes in future research.

## 1. Introduction

Endometriosis is a chronic disease in women of reproductive ages with symptoms of irregular menstruation, dysmenorrhea, and infertility [1]. The main pathological finding in endometriosis is ectopic endometrial tissue in the abdominal cavity. Several mechanisms of endometriosis have been proposed, including retrograde menstruation, transformation of the coelomic epithelium to ectopic endometrial tissue, and the stem cell theory [1,2,3,4]. These mechanisms have been elucidated through molecular and biological experiments using cell lines, animal models, and human samples.

Recently, with the development of high-throughput technologies for capturing molecular signals that occur during gene expression, various omics technologies have been used in translational research, including endometriosis research [5]. From genome-wide association studies (GWAS) detecting sequence variations that are prevalent in diseases to metabolomics, the identification of metabolites in patient samples has been applied to endometriosis research. Several genes have been associated with endometriosis. Unlike traditional molecular biological studies, translational research using genomic technologies tends to have many genes in a single experiment; it is important to characterize the gene list to understand the underlying biological mechanisms of a disease.

In this review, the results of endometriosis research using high-throughput technologies are discussed to determine whether they are consistent with the molecular mechanisms of endometriosis identified through independent molecular biology studies. For this purpose, the biological semantics of endometriosis were identified using a disease-related gene database and gene ontology enrichment analysis. Genes obtained from omics technologies were investigated to determine whether they represented the underlying biological semantics of endometriosis. The results from omics technologies were also applied to enrichment analysis and the consistency of their results was compared. The omics data focused on GWAS, methylomics, and transcriptomics, which are directly related to gene expression. In addition, studies comparing normal endometrium and endometrial lesions were also considered.

## 2. Biological Characteristics of Endometriosis

To date, several genes have been found to be associated with the pathophysiology of endometriosis. In many cases, these genes have been identified through molecular biology experiments. With the advent of high-throughput technologies, such as micro-array or next-generation sequencing (NGS), several endometriosis-related genes have been discovered in a single experiment. Before discussing the performance of high-throughput technologies, the biological mechanisms involved in endometriosis were summarized using over-representation analysis (ORA). ORA is a gene set method that tests the non-random appearance of genes in a gene set in an input list of genes [6]. The test results indicated that the tested gene list contained more genes in a gene set than what was expected. In this test, gene sets with functional meaning, such as gene ontology, gene modules, or pathways, were used. Fisher’s exact test was used for over-representation (Supplementary Methods). Significant *p* value of the enrichment analysis with Fisher’s exact test indicated that genes of a functional gene set were more included in the input gene list than randomly expected. If some gene sets have hundreds or thousands of genes, it is possible that substantial amounts of genes of the gene set were included in the randomly sampled genes. For example, if the gene set had one-third of whole genes in a genome, about one-third of genes that were randomly sampled from the genome were very likely to be the genes of the gene set. That is, if the result of enrichment analysis was significant, it meant that genes of the gene set were more included in the list than randomly expected. Consequently, the biological meaning of significant result was that the function of the gene set was associated with a specific condition or phenotype because more genes were included in the input genes. For example, if endometriosis disease genes were enriched with genes of a pathway gene set, it could be interpreted that the pathway was associated with the pathogenesis of endometriosis. Significant terms and pathways were used as reference biological characteristics or concepts for endometriosis.

**Figure 1:**
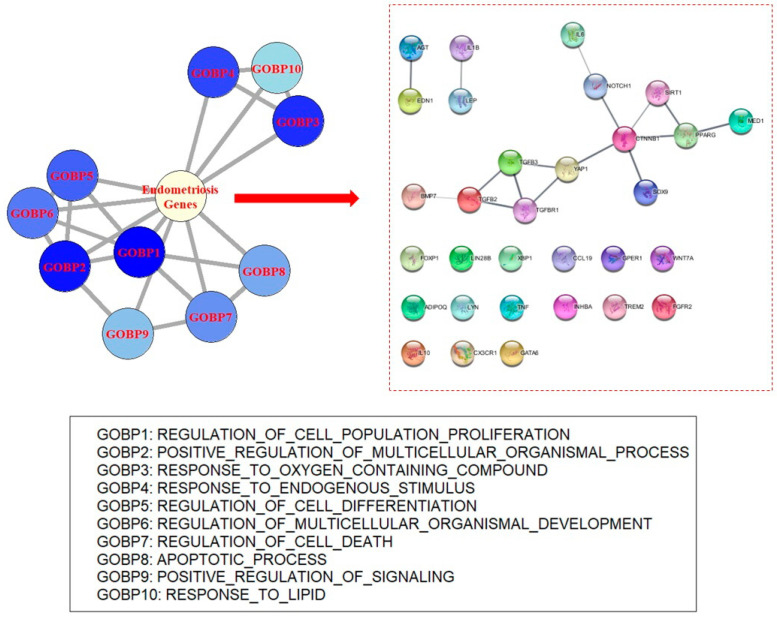
Gene ontology network of endometriosis disease genes. In this figure, the top 10 gene ontological process terms from the enrichment study of endometriosis-associated genes and their network structure are visualized (see Supplementary Methods for network determination). The highlighted network shows an interaction between genes included in endometriosis-related genes and common genes of top-ranked gene ontology members. Note that *CTNNB1* and *YAP1* of the highlighted network seem to be hub genes. According to current knowledge, 15 genes have no determined physical associations. See Supplementary Methods for determination of network edges. GOBP—gene ontology biological process.

To identify the central concept of endometriosis, ORA was applied to disease-related genes involved in endometriosis. Data regarding endometriosis-related genes were obtained from the DisGeNET database [7]. In the database, 1188 genes that were known to be associated with endometriosis were retrieved. Genes with no specific publications concerning their association with endometriosis were excluded (*n* = 4). Genes identified in omics data analysis were also removed (*n* = 29). A total of 1155 genes were used for the analysis (Table S1). Gene ontology biological process (GOBP) terms and Kyoto Encyclopedia of Genes and Genomes (KEGG) pathway gene sets were used for ORA [8,9]. Many GOBPs and KEGG pathways were found to be significant, with adjusted *p* value thresholds (Supplementary Methods). In total, 2614 GOBP terms were significant and the top-ranked GOBPs indicated the pathophysiology of endometriosis. For example, “regulation of cell population proliferation” yielded the most significant result (odds ratio (OR) = 16.80, *p* value = 2.53 × 10^−283^). In addition, “regulation of multicellular processes”, “response to oxygen-containing compounds”, “response to endogenous stimuli”, and “regulation of cell differentiation” were observed in the top 10 most significant results (Table 1, Figure 1 and Table S2). GOBPs were involved in canonical biological processes in endometriosis.

The results of ORA using KEGG pathways revealed a large number of pathways related to endometriosis. Of the 186 pathways, 97 were significant (Table 2 and Table S3). These pathways were consistent with the pathophysiology of endometriosis. In the top-ranked results, “cytokine receptor interaction”, “chemokine signaling pathway”, and “focal adhesion” pathways were included; these pathways are well-known mechanisms involved in endometriosis. Interestingly, cancer pathways were highly significant, regardless of the type of cancer. Oncogenic pathways specific to different cancers were defined separately in the KEGG database. In the top-ranked results, many cancer pathways involving prostate, pancreas, hematopoietic system, bladder, lower digestive system, skin, lungs, and brain were significant. For example, the “pathways in cancer” showed the most significant result (OR = 24.31, *p* value = 1.89 × 10^−105^). Moreover, “prostate cancer”, “pancreatic cancer”, “chronic myeloid leukemia”, and “colorectal cancer” pathways were listed in the top 10 significant results (Table 2).

## 3. Genome-Wide Association Study with SNP Micro-Array and Next Generation Sequencing

To identify sequence variants that affect the development of endometriosis, association studies of genetic variants, especially SNPs, have been actively performed. With the advent of high-throughput SNP microarrays and NGS, many SNPs can be examined in a single experiment. Unfortunately, most SNPs have small or moderate effect sizes; hence, significant results are difficult to obtain in small-scale studies. Therefore, large population cohorts and meta-analyses are frequently used to identify significant results [10].

To identify the biological mechanisms determined using GWAS, the results of endometriosis GWAS were retrieved from the GWAS catalogue database [11]. When a phenotype term was set to “EFO_0001065”, indicating endometriosis, there were 391 variants involving 307 genes from 21 studies (Table S4). Of the 307 genes, 38 overlapped with a list of endometriosis-related genes. ORA with GOBP showed 60 significant results, of which 59 were listed in the ORA results for endometriosis disease genes (Table 3 and Table S5). These GOBP terms included “regulation of locomotion” (OR = 4.26, *p* value = 2.00 × 10^−10^), “cell adhesion” (OR = 3.65, *p* value = 4.85 × 10^−10^), and “MAPK cascade” (OR = 4.40, *p* value = 1.85 × 10^−8^), which are relevant to the pathophysiology of endometriosis. For example, the GOBP term “regulation of locomotion” includes 31 genes that were identified through GWASs, such as *WNT4*, *IGF1R*, and *ERBB4*. The rs2235529 SNP of *WNT4* is associated with the development of endometriosis in European populations [12]; additional validation studies have replicated the association of *WNT4* with endometriosis [13,14,15]. *IGF1R* was found in a GWAS of the Chinese population [16] and has been associated with estrogen receptor expression [17]. An association between *ERRB4* and endometriosis was found in a study regarding the comorbidity between endometriosis and migraine [18]. Although the role of *ERRB4* has not been revealed in functional studies concerning endometriosis, it may play a role in estrogen-related gene regulation [19].

“Cell adhesion” is another key biological process involved in the pathophysiology of endometriosis. Cell adhesion molecules promote the binding of ectopic endometrial tissues to target organs [20]. In total, 23 genes that belong to the “cell adhesion” GOBP term have been identified as disease genes for endometriosis. These genes include *BCL2*, *IGF1*, *IL1B*, *PTEN*, and *NRP1*. Stromal BCL2 levels are elevated in endometriosis [21,22] and GWAS-identified sequence variants of *BCL2* are associated with endometriosis [18]. While BCL2 is known to inhibit cell adhesion [23], up-regulation of BCL2 has been reported in endometrial tissues [24]. Considering the increased cell adhesion activity in endometriosis, the role of BCL2 in the cell adhesion mechanism in endometriosis should be clarified. IGF1 was found in a GWAS of endometriosis in East Asian and European populations [25]. IGF1 expression is elevated in patients with endometriosis [26] and IGF1 signaling is associated with endometrial regeneration [27,28]. IL1B has a role in the regulation of cell adhesion [29]. IL1R2, which encodes a receptor molecule for IL1, is over-expressed in ectopic endometrial tissues, which is implicated in aberrant endometrial cell responsiveness to implantation [30]. The elevation of IL1B in endometriotic cells is activated by estrogen receptor beta, which increases the adhesiveness of endometriotic lesions. [31].

The GOBP term “tube morphogenesis”, which is related to vascular development, was highly ranked in the results of enrichment analysis (OR = 4.01, *p* value = 1.27 × 10^−9^, Table 3). PTEN is well known for its angiogenic activity in cancer [32]. In endometriosis, the down-regulation of PTEN is associated with the up-regulation of VEGF that is a main angiogenic molecule [33,34]. GATA4 is involved in angiogenesis [35,36] and its angiogenic effect is associated with ovarian-like differentiation in endometriosis [37]. ETS1 is found to be related to angiogenesis, measured by correlation with microvessel count [38].

In the results of ORA, the GOBP term “neurogenesis” was significant. LAMC3 plays a role in neurogenesis; mutations in *LAMC3* can cause cortical malformation [39,40]. LAMC3 is up-regulated in the eutopic endometrium of endometriosis [41] and in menstrual mesenchymal stem cells [42]. Basigin (BSG; CD147) is required for maintaining the *Drosophila* visual system; abnormal BSG causes misplacement of glial cells and disruption of capitate projections between the glia and photoreceptors in *Drosophila*. CD147 is involved in epithelial-to-mesenchymal transition (EMT) and is associated with the perturbation of normal apoptosis in endometriosis [24,43,44]. However, there is no definitive evidence that CD147 is associated with abnormal neurogenesis in endometriosis. Because EMT is related to neurogenesis, it is possible that CD147 is associated with the formation of abnormal innervations in endometriosis.

ORA with GOBP revealed that GWAS hotspots of endometriosis include proliferation-related genes, resulting in significant GOBP terms associated with cell proliferation, growth, and development (Table 3 and Table S5). For example, 22 genes in the GOBP term “growth” were included in the GWAS results for endometriosis (Table S5). Of these genes, *BCL2* is a well-characterized proliferation-related gene involved in endometriosis. As mentioned earlier, BCL2 is involved in cell adhesion processes and, considering that cell adhesion and proliferation are associated [45], it is natural that BCL2 is involved in both these processes. In endometriosis, BCL2 is influenced by mir-148a [46] and CCL19/CCR7 via the PI3K/Akt signaling pathway [47]. BCL2 is also over-expressed in adenomyosis [48]. IGF1 is a well-known growth-promoting factor in cells [49]; its role in the proliferation of endometriotic cells has been elucidated [50,51,52,53]. The proliferative effect of IGF1 is mediated by the mTOR [51,53] and MAPK pathways [52]. IGF1 has been reported as a hub gene in the bioinformatic analysis of endometriosis transcriptomics data [54]. Differential expression of IGF1 is related to changes in the epigenetic profile or estrogen signaling.

The ORA with KEGG pathways revealed findings consistent with those of the endometriosis disease genes (Table 4 and Table S6). Although only seven pathways were significant in the ORA, all pathways were listed in the results for endometriosis disease genes. The “pathways in cancer” tag exhibited the most significant result, similar to the results obtained for endometriosis disease genes (Table 4). In addition to the “pathways in cancer”, there were many signaling pathways such as “GnRH signaling pathway”, “calcium signaling pathway”, and “phosphatidylinositol signaling pathway” (Table 4). Interestingly, the “long term depression” pathway was highly ranked, which is consistent with previous findings (Supplementary Figure S1).

## 4. Transcriptome Analysis of Endometriosis Using Micro-Array or RNA Sequencing

To identify the molecular mechanisms revealed via transcriptomic data using gene expression micro-array or RNA sequencing platforms, data were collected from PubMed [55]. For this purpose, keyword searches including “human”, “endometriosis”, “gene expression”, “microarray”, and “next-generation sequencing” were used. We used the first three as default keywords and, alternatively, “microarray” or “next-generation sequencing”. All keywords were entered with the AND Boolean operator. To retrieve single-cell-sequencing endometriosis-related research, the first three keywords and “single cell sequencing” were applied. To retrieve transcriptomics data, articles with results of DEGs between normal endometrium and endometriosis lesions were used. For methylomics research, “human”, “endometriosis”, and “methylation” were used as default keywords, and, alternately, “microarray”, and “next-generation sequencing”. Of the articles identified by the PubMed search, only those with a list of genes differentially expressed between normal endometrium and endometriotic lesions were used for this review. Only the publications with a list of differentially expressed genes (DEGs) between normal tissues and endometriotic lesions were included. In total, 15 datasets having transcriptomic data on endometriosis were collected (Table S6). A list of DEGs was collected, and redundant genes were removed. Consequently, 3761 genes were identified as DEGs (Table S7); of these, 417 genes overlapped with endometriosis-related genes from the DisGeNet database.

When the 3761 genes were subjected to enrichment analysis, substantial concurrent results with those of endometriosis disease genes were obtained. In the ORA with GOBP, 1910 terms were significant (Table 5 and Table S8) and 1635 terms overlapped with significant results of the ORA with GOBP for endometriosis disease genes. Of the significant GOBP terms, “immune response” showed the most significant result (OR = 5.32, *p* value = 7.54 × 10^−179^). In total, 593 genes were identified from the results of transcriptome data analysis.

C3 is a member of the complement system, which is involved in the innate immune system. In endometriosis, C3 protein and gene expression are elevated [56,57,58] and ectopic endometrial tissue expresses C3, which results in the activation of TNF-alpha secretion by mast cells and enhancement of endometriosis development [59]. Notch1 is a crucial regulator of T cell responses [60]. When Notch signaling is blocked, endometriosis progression is inhibited by an increase in T helper cells and a decrease in regulatory T cells [61]. Fas is a subtype of tumor necrosis factor that plays a role in removing infected cells and dangerous lymphocytes through apoptosis [62]. Endometriosis shows reduced expression of Fas and increased expression of Fas ligand, which are related to the bypass of immune responses [63]. Of the genes of the GOBP term “immune response”, there are many interleukin-related genes, including *IL1B*, *IL6*, *IL4*, *IL7*, and *IL15*. It is well known that cytokines have roles in the pathogenesis of endometriosis [64]. Polymorphisms in IL1B have been associated with severe endometriosis [65]. IL1B simulates expression of migratory inhibitory factor with NFkappaB, which is believed to be involved in immune modulation [29]. In endometriosis, IL6 secretion is activated by IL1B, TNF-alpha, or hypoxia [66]. In a mouse model, IL6 plays a role in the development of early endometriosis with estrogen receptor-alpha [67]. The concentration of IL6 in the peritoneal fluid of patients with endometriosis is elevated and is correlated with infertility [68]. IL4 expression is up-regulated in endometriosis and is associated with alterations in Th2 immune modulation [69,70]. IL7 and IL15 have also been associated with endometriosis. Human studies have revealed that IL7 and IL15 are up-regulated in endometriosis [71,72] and are involved in suppressing natural killer cell activity [73,74].

The GOBP term “defense response” was also highly significant (OR = 5.21, *p* = 8.10 × 10^−164^). These terms include genes that function in defense mechanisms and are involved in responses to micro-organisms, tumor cells, and foreign bodies. Of the endometriosis genes identified using transcriptomic data, 551 were found to be members of the GOBP term “defense response”. SRC is a tyrosine protein kinase and a proto-oncogene. SRC has a role in mediating inflammatory responses via macrophages, which is accomplished by various signaling molecules, such as MAPK and FAK [75]. *COX2*, a gene involved in inflammatory responses, produces prostaglandin E2 and activates matrix metalloproteinases via SRC kinases [76]. SMAD3, a marker of EMT, is closely related to inflammation and is down-regulated in ectopic endometrium [77]. SMAD3 is associated with macrophage polarization, which is altered during endometriosis [78]. Since the “defense response” is related to inflammation, several chemokine ligand genes appear frequently in the results of transcriptomics data analysis. For example, CXCL1 is upregulated in endometriosis and is induced by E-Selectin and IL17A [79,80,81]. *CXCL12* transcription is up-regulated in endometriosis and is involved in the migration of endometriotic lesions [79,80,82,83]. CXCL10 of endometrial stromal cells is produced by hormone withdrawal with nuclear translocation of NFkappaB [84]. Toll-like receptors affect CXCL10 expression in the ectopic endometrium [85]. The expressions of CXCL13, CXCL14, CXCL15, and CXCL5 is altered in endometriosis, but their functional roles have not been clearly defined [86,87,88,89]. CCL2 is the most frequently studied chemokine involved in endometriosis. It is elevated in the peripheral blood and peritoneal fluid of patients [90,91]. Estrogen receptor and estradiol induce production of CLL2 via NFkappaB signaling and the p38 MAPK pathway [92,93,94]. Experimental results of therapeutic agents such as FR 167653, luteolin, and resveratrol revealed altered expression of CCL2 and other inflammatory molecules in endometriosis, indicating the therapeutic potential of these chemicals [95,96,97,98].

In the ORA, the GOBP term “apoptotic process” was highly significant (OR = 4.16, *p* value = 3.24 × 10^−120^). As abnormal changes in the apoptotic process occur in cancer tissues, endometriotic lesions also exhibit aberrant changes in the apoptotic process, according to the results of transcriptomic data analysis. TNF has a role in the destruction and regeneration of endometrial tissues in response to hormones and elevated levels of TNF are observed in peritoneal macrophages and endometriotic lesions [99]. TNF perturbs the development of mouse embryos and the inhibition of TNF reduces embryotoxic effects of endometriotic peritoneal fluid [100]. TNF appears to be involved in the establishment of ectopic lesions by interacting with KLF9, Notch, and the Hedgehog signaling pathways [101]. TNF-alpha-mediated induction of apoptosis in endometriosis can be blocked by the inhibition of DAK1 with mir-191 [102]. TNF-alpha-related apoptosis is also inhibited by estrogen receptor beta [31], which also affects the apoptosis of epithelial cells during endometriosis through phosphorylation of IkappaB kinase [103]. BCL2 is well-known for its regulatory role in the apoptosis of endometriosis. In endometriosis, the up-regulation of BCL2 decreases apoptosis, which is then increased by GnRH agonists [21,104,105,106,107]. CXCL8 increases BCL2 expression, which is related to increased survival of endometriotic lesions [106,108]. Activated ERK signaling and CD147 also up-regulate BCL2 [24]. Several non-coding miRNAs are involved in regulating BCL2 expression. Mir-196b affects *c-Myc* and *BCL2* mRNA expression [109]. Hsa_circ_000843, a circular RNA, inhibits the expression of BCL2, CDKN1B, and Cyclin D1 in endometriotic lesions [110]. The lncRNA *MALAT1* inhibits apoptosis of endometriosis cells via the PI3K-AKT pathway [111]. Mir-139-5p and BCL2 binding component 3 (BBC3) are down-regulated in ectopic endometrial tissues; inhibitor of mir-139-5p shows significantly decreased cell viability in endometriosis tissues [112]. LncRNA *AFAP-AS1* is involved in the conversion of E-cadherin to N-cadherin and expression of Snail and in activating the STAT3/TGFbeta1/Smad2 axis through directly inhibiting miR-424-5p [112]. *BAX* is an apoptosis-related gene that shows lower expression in peritoneal macrophages of patients with endometriosis [113] and exhibits higher expression in endometriomas than in other benign tumors [114]. *BAX* mRNA decreases in the endometriotic tissue compared with normal endometrium [107,115] and increases after GnRH agonist treatment [105]. *BAX* mRNA levels are decreased in endometriotic tissue compared with those in normal endometrium [116]. *APEX1* and miR-24 are highly expressed in endometriotic lesions; silencing of these genes elevates BAX expression [117]. CPEB3 increases BAX expression in endometriosis with decreased expression of MMP-2 and MMP-9, which are related to cell mobility and adhesion [118]. c-Myc is another important molecule in the apoptotic process that is up-regulated in endometriosis [115]. Stimulation of estrogen receptor beta up-regulates c-Myc, which is associated with G2/M cell cycle transition [119]. miR-196b and miR-488 are associated with the expression of c-Myc and other molecules that play roles in the apoptotic process in endometriosis [109,120]. Caspase-1, also called interleukin 1 converting enzyme (ICE), exhibits higher levels in endometriosis [121]. In endometriotic lesions, astrocyte elevated gene-1 (AEG-1) decreases the cleaved forms of Caspase-1 and SOCS1, promoting the formation of the NALP3 inflammasome [122,123]. Indeed, Caspase-1 and interleukin 1-beta are mediators of pyroptosis and are regulated by TRIM24 in endometriosis [124,125].

The GOBP term “response to cytokine” showed a highly significant enrichment result (OR = 6.30, *p* = 5.44 × 10^−119^). TNF-alpha is a cytokine that plays important roles in the growth and proliferation of endometriotic tissues [126]. TNF is positively correlated with C-reactive protein and VEGF in endometriosis patients [127]; it increases MMP-1, MMP-3, MMP-9, and ICAM1 levels, which are required for tissue invasion by endometriotic cells [128,129]. Serum or peritoneal fluid TNF levels are associated with infertility [130,131,132]. Serum TNF levels are inversely correlated with estradiol levels and decrease when patients are pregnant [130].

The results of the ORA with KEGG pathways showed that cytokine and signaling pathways were highly ranked (Table 6 and Table S9). In total, 90 pathways were significant with adjusted *p* values; 79 of the 90 pathways were also significant in the ORA of endometriosis disease genes. As in the result of ORA of endometriosis disease genes and GWAS genes, “pathways in cancer” showed the most highly ranked significance (OR = 5.44, *p* value = 1.47 × 10^−36^).

## 5. Single Cell Sequencing Analysis of Endometriosis

Single-cell sequencing methods include DNA sequencing of the genome, methylome, or transcriptome of a single cell [133]. Because omics data are generated at the single-cell level, it is useful to obtain large amounts of data from a small number of samples. Moreover, it is convenient for differentiating between heterogeneous cell types that are mixed in clinical samples [134]. For example, single-cell sequencing research has revealed cancer cells and their relationships with infiltrating immune cells. Moreover, revealing the composition of pathological lesions is relatively easy to accomplish using single-cell sequencing.

Considering that endometriotic lesions are prone to merge with immune cells and the importance of interactions with immune cells in the pathophysiology of endometriosis, single-cell technologies are expected to have a large impact on molecular studies based on a whole-genome scale. To date, only three publications have reported the results of single-cell RNA sequencing data from clinically sampled endometriotic lesions having normal and endometrial lesions [135,136,137]. This number is expected to increase because of the efficiency and capability of single-cell sequencing technologies. All studies provided DEG lists between endometrial and normal endometrium and genes in the list were obtained. A total of 3092 genes were differentially expressed in endometriosis (Table S10). Of these genes, 327 overlapped with endometriosis disease genes.

Enrichment analysis revealed that 1517 GOBP terms were significant; 1217 terms (80% of significant results) overlapped with those from the enrichment analysis of disease genes (Table 7 and Table S11). Regulation of cell death was the most significant GOBP term (OR = 4.95, *p* = 1.87 × 10^−125^). Among genes of the term, some were newly identified in single-cell RNA sequencing data. For example, the haptoglobin (*HP*) gene is involved in the regulation of cell death and was not found in transcriptome data analysis, whereas single-cell sequencing data found it to be differentially expressed in endometriosis. Endometriotic lesions secrete haptoglobins that adhere to peritoneal macrophages and prevent their phagocytic function [138]. Plasma haptoglobin levels are decreased in patients with endometriosis [139]. Inflammatory cytokines mediate haptoglobin in endometriosis [140]. The androgen receptor (*AR*) is another gene found only in single-cell sequencing data. It is well known that androgens and androgen receptors are associated with endometriosis. Genetic variants of the *AR* are associated with the development of endometriosis [141,142,143]. Moreover, androgens are associated with endometrial apoptosis [144], pain caused by endometriosis [145], and endometrioma [146]. In addition to “regulation of cell death”, several terms, such as “cell motility”, “cell adhesion”, “defense response”, and “immune response”, which are relevant to endometriosis pathophysiology, were highly significant GOBP terms (Table 7).

The results of enrichment analysis with KEGG pathway gene sets also showed highly overlapping results with those of endometriosis genes (Table 8 and Table S12). Among the significant KEGG pathways (*n* = 75), 80% (*n* = 60) also appeared in the significant results of enrichment analysis with endometriosis genes. The “Pathways in cancer” KEGG gene set exhibited the most significant result in the enrichment analysis of endometriosis genes; the same pathway showed a highly significant result in the enrichment analysis with genes of single-cell RNA sequencing analysis (OR = 4.04, *p* value = 1.89 × 10^−20^). Of the genes in the “pathways in cancer”, some genes were not in the list of endometriosis genes or genes from the analysis of transcriptomic data but were present in the analysis results of single-cell RNA sequencing data. For example, the expression of GRB2 and Grb2-associated binding protein 2 (GAB2) was elevated during endometriosis [147,148]. Pathways associated with neurodegenerative diseases, such as Alzheimer’s, Parkinson’s, and Huntington’s diseases, were significant in the enrichment analysis, similar to results from enrichment analysis of endometriosis genes (Table 8).

## 6. Methylome Analysis of Endometriosis

The epigenetic control of gene expression is one of the core processes related to disease development and progression. Abnormal epigenetic regulation has been reported in endometriosis. Among the various epigenetic mechanisms that regulate gene expression, DNA methylation has been identified using micro-arrays and NGS. Because methylation is a necessary epigenetic condition for gene expression, an abnormal methylation status indicates that genes affected by methylation are associated with endometriosis.

Although there have been numerous studies on transcriptomics in endometriosis, few have investigated genome-wide methylation using human samples. Five studies were available for analysis (Table S13). When genes that showed differential methylation status compared with normal controls were collected, 169 genes with abnormal methylation were identified (Table S14). From the results of the abovementioned enrichment analyses, many significant results were obtained after multiple testing corrections, although the number of GOBP terms or pathways was far less than that obtained in previous results, which resulted from a relatively small number of genes being involved. Moreover, these results were consistent with those of previous studies (Table 9 and Table S15). Among the 137 significant GOBP terms in the enrichment analysis, 135 overlapped with the significant results of enrichment analysis of endometriosis genes. Although overlapping GOBP terms were identified, the different GOBP terms were highly ranked. Of the significant GOBP terms, “negative regulation of RNA metabolic processes” was top-ranked (Table 9, OR = 10.66, *p* value = 4.50 × 10^−27^). Additionally, many GOBP terms related to developmental processes were highly ranked. For example, in the top 10 significant results, GOBP terms included “animal organ morphogenesis”, “tissue development”, “embryo development”, and “epithelial development”.

In the term “negative regulation of the RNA metabolic process”, genes are involved in inhibiting chemical reactions with RNA. This term includes many genes that encode transcription factors that reduce or stop reactions involving RNA metabolism. From the endometriosis methylation data, the methylation of such genes is found to be dysregulated. For example, *KLF11* is a transcription factor included in the list of genes with abnormal methylation. KLF11 is down-regulated in the ectopic endometrium and appears to activate fibrosis induced by collagen, MMP3, and TGFβR1, which are transcriptionally repressed by KLF11 [149]. SMAD3 is an intra-cellular signal transducer associated with the pathophysiology of endometriosis. It is down-regulated in the ectopic endometrium [77], binds to promoter II of *p450* aromatase, and promotes *p450* transcription, which is activated by activin A [150]. SMAD3 is also involved in the control of beta-glycan shedding induced by activin A [151,152]. WT1 is a transcription factor that regulates the mammalian urogenital epithelium; cAMP, which induces decidualization, increases WT1 expression [153]. WT1-positive fibroblasts are associated with platelet-induced mesothelial–mesenchymal transition in endometriosis [154].

“Tissue development” is a GOBP term referring to genes related to tissue formation and maturation. In this term, WT1 and SMAD3 are included, which indicates that these genes may have regulatory connections with other developmental genes. *PAX8* is another gene in the GOBP term related to the pathophysiology of endometriosis. It plays a role in the development of the female genital tract and its expression increases in the normal peritoneum of endometriosis patients [155]. It is a sensitive marker of extra-genital endometriosis [156] and sero-mucinous tumors associated with endometriosis [157,158].

The six KEGG pathways that were significant in the enrichment study were found in the list of significant results of the KEGG pathway enrichment analysis of endometriosis genes (Table 10).

## 7. Conclusions

In this review, we summarized the results of analysis of high-throughput data involving endometriosis. When the results were compared by genes, reproducibility was moderate. However, in terms of gene set enrichment analysis, results of omics data analysis were consistent with those of endometriosis genes found in independent studies without omics data. These results indicate that omics data analysis provides meaningful information regarding disease-related genes that are involved in the development of endometriosis. In particular, genes that were found by omics data analysis had moderate statistical significance in not reaching the adjusted *p* values estimated by multiple testing corrections; gene set enrichment studies of the genes showed that functional categories of the genes were consistent with the independently identified genes. This indicates that omics data analysis plays an important role in identifying genes associated with endometriosis. Moreover, single-cell RNA sequencing data showed significant results because hundreds or thousands of single cells were sequenced, which increased the sample size in the analysis. Only three single-cell RNA sequencing studies were included, but the results showed consistency equivalent to that of other methods. Given this, single-cell sequencing technology may be a more efficient and useful tool for translational research surrounding endometriosis.

In conclusion, various omics technologies have substantial potential for identifying disease-related genes that cause endometriosis. Genes that belong to significant GOBP or KEGG pathways and their roles in endometriosis have not yet been investigated.

## Figures and Tables

**Table 1:** Top 10 results of over-representation analysis (ORA) with GOBP for endometriosis disease genes.

GOBP	OR	*p* Value
Regulation of cell population proliferation	16.80	2.53 × 10^−283^
Positive regulation of multi-celluar organismal process	15.92	9.92 × 10^−247^
Response to oxygen containing compound	14.71	8.69 × 10^−241^
Response to endogenous stimulus	14.59	4.00 × 10^−234^
Regulation of cell differentiation	14.02	4.07 × 10^−224^
Regulation of multi-cellular organismal development	14.95	1.31 × 10^−222^
Regulation of cell death	13.42	2.63 × 10^−218^
Apoptotic process	12.04	2.85 × 10^−212^
Positive regulation of signaling	12.42	2.00 × 10^−206^
Response to lipid	19.47	9.95 × 10^−206^

GOBP—gene ontology biological process; OR—odds ratio.

**Table 2:** Top 10 results of ORA with KEGG pathway for endometriosis disease genes.

KEGG Pathway	OR	*p* Value
Pathways in cancer	24.31	1.89 × 10^−105^
Cytokine–cytokin receptor interaction	29.37	9.63 × 10^−104^
Chemokine signaling pathway	20.64	8.86 × 10^−55^
Focal adhesion	18.33	1.06 × 10^−50^
Prostate cancer	36.91	4.70 × 10^−45^
Pancreatic cancer	47.37	8.02 × 10^−43^
Chronic myeloid leukemia	40.76	3.24 × 10^−40^
Natural killer cell mediated cytotoxicity	19.75	6.40 × 10^−39^
MAPK signaling pathway	11.06	1.81 × 10^−37^
Colorectal cancer	45.44	3.61 × 10^−37^

KEGG—Kyoto Encyclopedia of Genes and Genomes; OR—odds ratio.

**Table 3:** Highly significant GOBPs in the ORA of results from GWAS of endometriosis.

GOBP	OR	*p* Value
Regulation of locomotion	4.26	2.00 × 10^−10^
Locomotion	3.78	3.47 × 10^−10^
Cell adhesion	3.65	4.85 × 10^−10^
Tube morphogenesis	4.41	1.27 × 10^−9^
Tube development	4.02	1.40 × 10^−9^
Regulation of multi-cellular organismal development	3.60	1.97 × 10^−9^
Regulation of cell differentiation	3.42	2.48 × 10^−9^
Circulatory system development	3.82	4.13 × 10^−9^
Positive regulation of MAPK cascade	5.84	4.26 × 10^−9^
Central nervous system development	3.91	1.42 × 10^−8^

GOBP—gene ontology biological process; OR—odds ratio.

**Table 4:** Highly significant KEGG pathways in the ORA of results from GWAS of endometriosis.

KEGG Pathway	OR	*p* Value
Pathways in cancer	7.05	1.56 × 10^−8^
Long-term depression	15.85	7.02 × 10^−7^
Focal adhesion	7.64	1.79 × 10^−6^
GnRH signaling pathway	10.61	8.35 × 10^−6^
Small cell lung cancer	10.93	3.14 × 10^−5^
Calcium signaling pathway	6.72	4.48 × 10^−5^
Phosphatidylinositol singaling system	9.98	2.15 × 10^−4^

KEGG—Kyoto Encyclopedia of Genes and Genomes; OR—odds ratio; GnRH—gonadotropin-releasing hormone.

**Table 5:** Top 10 results of ORA with GOBP using result genes of endometriosis transcriptomics data.

GOBP	OR	*p* Value
Immune response	5.32	7.54 × 10^−179^
Regulation of immune system process	5.98	1.84 × 10^−171^
Defense response	5.21	8.10 × 10^−164^
Cell adhesion	5.67	6.76 × 10^−161^
Biological process involved in interspecies interaction between organisms	5.10	1.02 × 10^−151^
Positive regulation of multi-cellular organismal process	5.05	4.12 × 10^−137^
Cell activation	6.07	7.29 × 10^−137^
Tissue development	4.28	2.18 × 10^−130^
Cell motility	4.51	5.97 × 10^−130^
Response to oxygen containing compound	4.62	2.92 × 10^−128^

GOBP—gene ontology biological process; OR—odds ratio.

**Table 6:** Top 10 results of ORA with KEGG using genes of endometriosis transcriptomics data.

KEGG Pathway	OR	*p* Value
Cytokine–cytokine receptor interaction	11.29	1.82 × 10^−71^
Hematopoetic cell lineage	18.32	1.10 × 10^−36^
Pathways in cancer	5.44	1.47 × 10^−36^
Chemokine signaling pathway	7.53	1.15 × 10^−33^
Cell adhesion molecules CAMs	9.61	1.11 × 10^−31^
Leshimania infection	14.88	2.52 × 10^−26^
Toll-like receptor signaling pathway	10.24	2.56 × 10^−26^
Natural killer cell mediated cytotoxicity	7.37	2.46 × 10^−24^
JAK-STAT signaling pathway	6.37	7.68 × 10^−23^
Antigen processing and presentation	10.16	9.09 × 10^−23^

KEGG—Kyoto Encyclopedia of Genes and Genomes; OR—odds ratio.

**Table 7:** Highly significant GOBPs in the ORA of genes from endometriosis single-cell RNA sequencing data.

GOBP	OR	*p* Value
Regulation of cell death	4.95	1.87 × 10^−125^
Cell motility	4.77	3.18 × 10^−124^
Tissue development	4.50	7.47 × 10^−124^
Apoptotic process	4.57	1.14 × 10^−123^
Organonitrogen compound biosynthetic process	4.41	8.35 × 10^−111^
Cell adhesion	4.66	2.35 × 10^−105^
Response to oxygen containing compound	4.30	1.02 × 10^−99^
Biological process involved in interspecies interaction between organisms	4.23	1.48 × 10^−98^
Regulation of cell population proliferation	4.13	6.22 × 10^−97^
Response to endogenous stimulus	4.11	4.22 × 10^−90^

GOBP—gene ontology biological process; OR—odds ratio.

**Table 8:** Highly significant KEGG pathways in the ORA of genes from endometriosis single-cell RNA sequencing data.

KEGG Pathway	OR	*p* Value
Ribosome	280.52	3.24 × 10^−91^
Oxidative phosphorylation	22.59	3.75 × 10^−61^
Parkinson’s disease	19.34	5.47 × 10^−54^
Huntington’s disease	12.50	1.75 × 10^−51^
Alzheimer’s disease	12.09	1.08 × 10^−45^
Focal adhesion	7.58	2.33 × 10^−32^
Leukocyte transendothelial migration	8.78	3.13 × 10^−23^
Pathogenic Escherichia coli infection	16.27	2.43 × 10^−21^
Pathways in cancer	4.04	1.89 × 10^−20^
ECM receptor interaction	9.38	1.04 × 10^−18^

KEGG—Kyoto Encyclopedia of Genes and Genomes.

**Table 9:** Top 10 GOBPs identified using ORA as genes having methylation changes in endometriosis.

GOBP	OR	*p* Value
Negative regulation of RNA metabolic process	10.66	4.50 × 10^−27^
Negative regulation of nucleobase containing compound metabolic process	9.70	1.63 × 10^−25^
Animal organ morhogenesis	11.56	1.57 × 10^−24^
Negative regulation of biosynthetic process	8.77	7.07 × 10^−24^
Tissue development	7.64	4.40 × 10^−22^
Negative regulation of transcription by RNA polymerase II	11.07	1.07 × 10^−21^
Embryo development	9.97	1.99 × 10^−21^
Epithelium development	8.96	1.50 × 10^−19^
Pattern specification process	14.96	1.41 × 10^−18^
Positive regulation of RNA metabolic process	6.65	2.22 × 10^−17^

GOBP—gene ontology biological process; OR—odds ratio.

**Table 10:** Significant KEGG pathways identified using ORA as genes having methylation changes in endometriosis.

KEGG Pathway	OR	*p* Value
Pathways in cancer	12.40	5.09 × 10^−11^
Basal cell carcinoma	26.19	2.57 × 10^−6^
Small cell lung cancer	16.55	2.08 × 10^−5^
Wnt signaling pathway	10.88	3.07 × 10^−5^
Non-small cell lung cancer	20.82	6.15 × 10^−5^
Thyroid cancer	29.86	2.00 × 10^−4^

KEGG—Kyoto Encyclopedia of Genes and Genomes.

## Supplementary Materials

The following supporting information can be downloaded at: https://www.mdpi.com/article/10.3390/biomedicines11082210/s1, Supplementary Methods and Results [6,159,160,161]; Figure S1: Interaction map between genes common to endometriosis and the long-term depression KEGG pathway; Table S1: Total list of endometriosis disease genes from DisGeNet database; Table S2: All significant result of over-representation analysis (ORA) with GOBP for endometriosis disease genes; Table S3: All significant result of ORA with KEGG for endometriosis disease genes; Table S4: A list of SNPs from GWASs for endometriosis; Table S5: Total list of significant GOBPs in the ORA of results from GWAS of endometriosis; Table S6: Total list of significant KEGG pathways in the ORA of results from GWAS of endometriosis; Table S7: Differentially expressed genes from transcriptomics data of endometriosis; Table S8: Total significant results of ORA with GOBP using result genes of endometriosis transcriptomics data; Table S9: Total significant results of ORA with KEGG using result genes of endometriosis transcriptomics data; Table S10: Differentially expressed genes from single cell RNA-sequencing data of endometriosis; Table S11: All significant GOBPs in the ORA of genes from endometriosis single-cell RNA sequencing data; Table S12: All significant KEGG pathways in the ORA of genes from endometriosis single-cell RNA sequencing data; Table S13: Publication list of research with methylomics data of endometriosis; Table S14: Differentially methylated genes from analysis of methylomics data of endometriosis; Table S15: Total significant GOBPs identified using ORA as genes having methylation changes in endometriosis.
