# Supplementary material for: Molecular Mechanisms of Endometriosis Revealed Using Omics Data
Source: Biomedicines. 2023 Aug 7;11(8):2210. doi: 10.3390/biomedicines11082210 (PMC10452455; doi:10.3390/biomedicines11082210)
Supplement: Supplementary file 1 [file biomedicines-11-02210-s001.zip › Supplementary Methods and Results.pdf]

Supplementary Methods

Overrepresentation analysis

Overrepresentation analysis a gene set analysis method testing the enrichment for genes having the same biological implications [1]. For this analysis, Fisher's exact test was applied. After obtaining a list of disease genes, genes of gene ontologies (GOs) or pathways were identified in the disease genes. ORA identifies whether more genes of a GO or pathway are included in the list than the number of the GO or pathway genes that are included randomly.

	Disease gene (+)	Disease gene (–)	
Gene set genes (+)	*a*	*b*	*a*+*b*
Gene set genes (–)	*c*	*d*	*c*+*d*
	a+*c*	*b*+*d*	*a*+*b*+*c*+*d* (= *n*)

In the above table, the positive sign indicates that a gene is included in the list; the negative sign means the gene is not. *n* indicates the total number of human genes. Fisher's exact test identifies whether the distribution of numbers in the table results from random chance. The P-value of the test is obtained using the hypergeometric distribution, as shown in equation (1).

$$P = \frac{(a+b)!(c+d)!(a+c)!(b+d!)}{a!b!c!d!n!} \qquad (1)$$

In the ORA, a one-sided test was used because the alternative hypothesis is that more genes of a functional gene set (GO or pathway) are included in the list than by random chance. The fisher.test function of the R program was applied, and it was set to

perform one-sided test.

Collection of gene sets

The information about gene ontology biological process (GOBP) terms and Kyoto Encyclopedia of Genes and Genomes (KEGG) pathways was obtained from gene set enrichment analysis (GSEA) website (https://www.gsea-msigdb.org/gsea/index.jsp). In total, 7763 GOBPs and 186 KEGG pathways were collected. The number of genes in GOBPs ranges from 5 to 1998 (mean = 83.39). The mean number of KEGG pathway genes was 68.80. For reference genes, 43161 genes were collected from HUGO Gene Nomenclature Committee (HGNC, https://www.genenames.org/)

Gene ontology network determination

For network visualization of gene ontologies, the density of node colors was determined according to the –log(*p* value). If *p* values were highly significant, then the node colors became denser. Edges were determined according to the Jaccard index, which is determined by the number of genes in common with and at the union between two gene ontologies. Genes with greater than the 75th quartile of all possible Jaccard indices were treated as positive edges. Edges between endometriosis genes and GOBPs were placed regardless of Jaccard index values.

Supplementary Results

Supplementary Figure S1

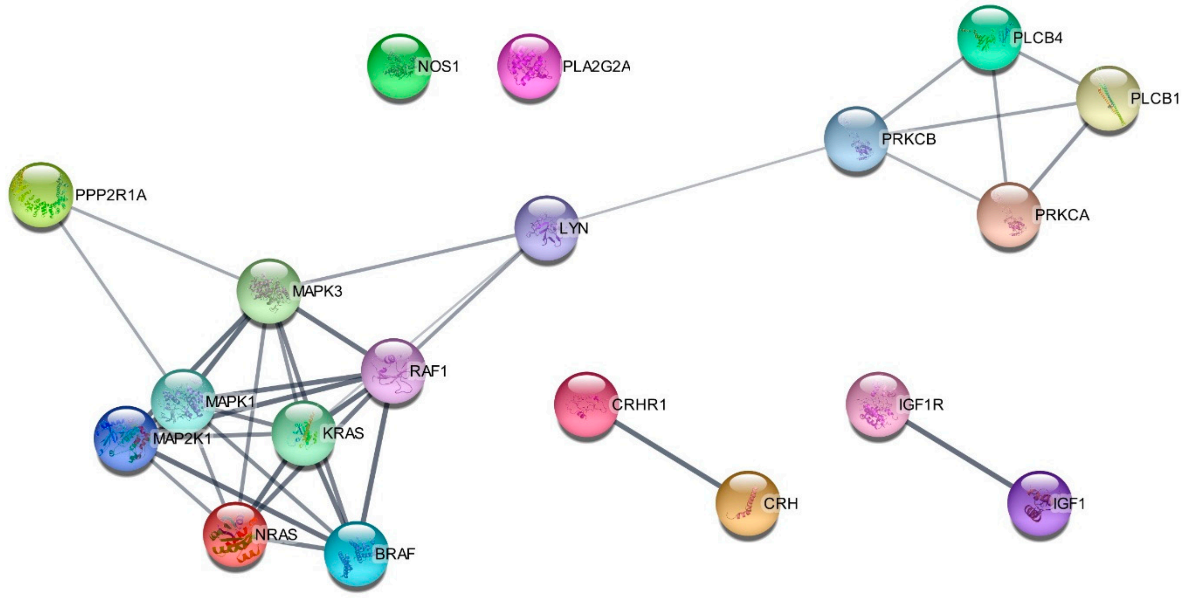

Supplementary Figure S1. Interaction map between genes common to endometriosis and the long-term depression KEGG pathway. Since comorbidity between depression and endometriosis is well known [2–4], common genes between the pathway and endometriosis disease genes were visualized to identify core genes of the comorbidity. The common genes were applied to the STRING database, and physical interactions of the genes were visualized. There were 19 genes in common between endometriosis and the long-term depression KEGG pathway. Denser edges indicate stronger evidence of interactions between the genes, which are represented by the edges. Some genes show tight interconnections and clusters, whereas others have no edges with other genes. *LYN* and *PRKCB* can be subordinated genes of the network, indicating their functional importance in the development of both diseases.

Reference

1. Maleki F, Ovens K, Hogan DJ, Kusalik AJ. Gene Set Analysis: Challenges, Opportunities, and Future Research. Front Genet. 2020 Jun 30;11:654. doi: 10.3389/fgene.2020.00654. PMID: 32695141; PMCID: PMC7339292.

2. Laganà AS, La Rosa VL, Rapisarda AMC, Valenti G, Sapia F, Chiofalo B, Rossetti D, Ban Frangež H, Vrtačnik Bokal E, Vitale SG. Anxiety and depression in patients with endometriosis: impact and management challenges. Int J Womens Health. 2017 May 16;9:323-330. doi: 10.2147/IJWH.S119729. PMID: 28553145; PMCID: PMC5440042.

3. Li T, Mamillapalli R, Ding S, Chang H, Liu ZW, Gao XB, Taylor HS. Endometriosis alters brain electrophysiology, gene expression and increases pain sensitization, anxiety, and depression in female mice. Biol Reprod. 2018 Aug 1;99(2):349-359. doi: 10.1093/biolre/ioy035. PMID: 29425272; PMCID: PMC6692844.

4. Estes SJ, Huisingh CE, Chiuve SE, Petruski-Ivleva N, Missmer SA. Depression, Anxiety, and Self-Directed Violence in Women With Endometriosis: A Retrospective Matched-Cohort Study. Am J Epidemiol. 2021 May 4;190(5):843-852. doi: 10.1093/aje/kwaa249. PMID: 33184648; PMCID: PMC8247611.
